# Respiratory impedance in healthy unsedated South African infants: Effects of maternal smoking

**DOI:** 10.1111/resp.12463

**Published:** 2015-01-11

**Authors:** Diane Gray, Dorottya Czövek, Emilee Smith, Lauren Willemse, Ane Alberts, Zoltán Gingl, Graham L Hall, Heather J Zar, Peter D Sly, Zoltán Hantos

**Affiliations:** 1 Department of Paediatrics and Child Health, Red Cross War Memorial Children's Hospital, University of Cape TownCape Town, South Africa; 2 Department of Medical Physics and Informatics, University of SzegedSzeged, Hungary; 3 Queensland Children's Medical Research Institute, University of QueenslandBrisbane, Queensland; 4 Center for Infectious Disease Epidemiology and Research, University of Cape TownCape Town, South Africa; 5 Department of Technical Informatics, University of SzegedSzeged, Hungary; 6 Telethon Kids Institute, University of Western AustraliaPerth, Western Australia, Australia

**Keywords:** forced oscillation technique, respiratory compliance paediatric, respiratory function test, respiratory resistance

## Abstract

**Background and objective:**

Non-invasive techniques for measuring lung mechanics in infants are needed for a better understanding of lung growth and function, and to study the effects of prenatal factors on subsequent lung growth in healthy infants. The forced oscillation technique requires minimal cooperation from the individual but has rarely been used in infants. The study aims to assess the use of the forced oscillation technique to measure the influence of antenatal exposures on respiratory mechanics in unsedated infants enrolled in a birth cohort study in Cape Town, South Africa.

**Methods:**

Healthy term infants were studied at 6–10 weeks of age using the forced oscillation technique. Respiratory impedance was measured in the frequency range 8–48 Hz via a face mask during natural sleep. Respiratory system resistance, compliance and inertance were calculated from the impedance spectra.

**Results:**

Of 177 infants tested, successful measurements were obtained in 164 (93%). Median (25–75%) values for resistance, compliance and inertance were 50.2 (39.5–60.6) cmH_2_O.s.L^−1^, 0.78 (0.61–0.99) mL.cmH_2_O^−1^ and 0.062 (0.050–0.086) cmH_2_O.s^2^.L^−1^, respectively. As a group, male infants had 16% higher resistance (*P* = 0.006) and 18% lower compliance (*P* = 0.02) than females. Infants whose mothers smoked during pregnancy had a 19% lower compliance than infants not exposed to tobacco smoke during pregnancy (*P* = 0.005). Neither maternal HIV infection nor ethnicity had a significant effect on respiratory mechanics.

**Conclusions:**

The forced oscillation technique is sensitive enough to demonstrate the effects of tobacco smoke exposure and sex in respiratory mechanics in healthy infants. This technique will facilitate assessing perinatal influences of lung function in infancy.

**SUMMARY AT A GLANCE**

Knowledge of the impact of prenatal exposures (such as maternal smoking) on infant lung function has been limited by difficulties with measuring lung function in healthy infants. This study uses a non-invasive method for the measurement of respiratory system impedance at a high success rate in a healthy infant cohort.

## Introduction

The assessment of lung mechanics in healthy infants offers the potential to better understand normal lung growth and function, the determinants of early lung development, and the relationship between lung function and respiratory disease. The forced oscillation technique (FOT) is a promising tool for measurement of lung function in infants as it is non-invasive, versatile and does not require controlled respiratory manoeuvres.^1^ Moreover, the small-amplitude oscillations are superimposed on spontaneous breathing, so measurements can be taken without interfering with normal respiration.

By imposing an external driving signal on the respiratory system and recording its response, the FOT directly measures the mechanical impedance of the respiratory system (Zrs). Zrs consists of the respiratory resistance (Rrs) and reactance (Xrs). Rrs is associated with the frictional losses and may be used as a surrogate for the airway resistance. Xrs expresses the balance between the elastic forces of the respiratory tissues and the inertial forces of the large airways dominating at the lower and higher frequencies, respectively.

As a consequence of the non-invasive nature and the minimal demand for cooperation, the FOT has gained popularity in paediatric lung function testing, and several coherent normative datasets have been published.^2^,^3^ However, its use in infancy has been sporadic and largely confined to methodological validation studies,^4^–^13^ all using sedation except one.^11^ The development of an FOT approach that is able to non-invasively measure respiratory system impedance (Zrs) in infants during natural sleep would provide the opportunity to track the mechanical properties of the lung through the early years of life, a time of critical lung growth and development. In addition to the establishment of normative data in infancy, such a method would be useful in the studies on effects of prenatal factors that may impact on later respiratory health, such as maternal smoking and HIV infection, the prevalence of which is high in South African populations.

The purpose of this study was therefore to (i) describe the mechanics of the respiratory system using FOT in healthy unsedated infants, and (ii) assess the impact of antenatal and early life factors on respiratory mechanics in infants from a large birth cohort study in a low- middle-income setting in South Africa.

## Methods

All infants enrolled in the Drakenstein Child Health Study, a birth cohort study established in a peri-urban area outside Cape Town, South Africa, had infant lung function tested at 6–10 weeks and planned annually from 1 to 5 years. For the purpose of the current study, all premature infants (born at <37 weeks) and those who had previous lower respiratory tract infection were excluded. The study population are of African ancestry, enrolled from health clinics serving two predominantly low socioeconomic communities. Details of the study population and setting have been published,^14^ and are described in the online supporting information. The study was approved by the Faculty of Health Sciences, Human Research Ethics Committee, University of Cape Town (401/2009) and by the Western Cape Provincial Health Research Committee. Mothers gave informed, written consent in their first language for their infants to participate.

### Measurement of lung function

**Figure 1:**
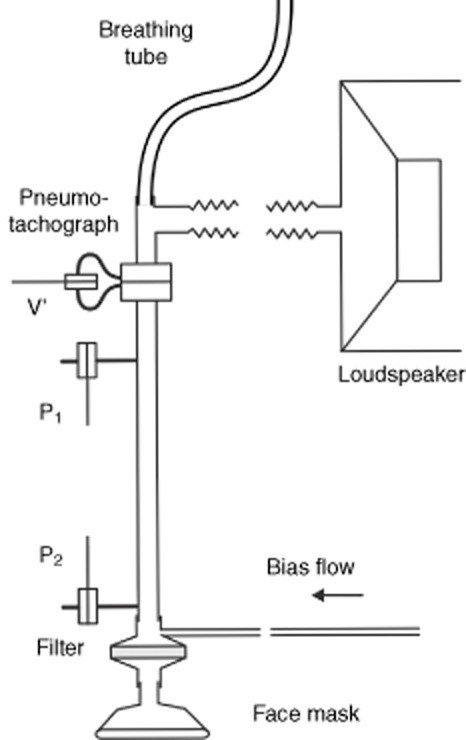
Schematic representation of the forced oscillation equipment. A loudspeaker delivers the computer-generated multicomponent forcing function. Pressure is measured at each end of a wave-tube (P1 and P2) for the estimation of respiratory impedance, and a pneumotachograph is used to monitor tidal airflow (V'). A bias flow reduces the influence of equipment dead-space on the infant's breathing pattern.

Zrs was measured with purpose-built FOT equipment (Fig. 1). A composite driving signal (frequencies: every 4 Hz between 8 and 48 Hz, pressure amplitude: <1 cmH_2_O) was generated by a loudspeaker and delivered to the infant through a wave-tube (internal diameter: 1 cm), an anti-bacterial filter (Humid-Vent, No. 19502, Teleflex Medical, Athlone, Ireland) and a face mask (Neonate Crystal Anaesthesia Mask, No. 39170, Koo Asia, Hong Kong). The inlet and outlet pressures of the wave-tube were sensed by ICS transducers (Model 33NA002D, ICSensors, Milpitas, CA, USA). Zrs was calculated as the load impedance on the tube,^15^ after corrections for the equipment impedances of the filter and the face mask. A pneumotachograph with a differential pressure transducer (ICS model 33NA002D) was attached to the wave-tube for monitoring the infant's breathing pattern. The dead space of the equipment was continuously flushed by a bias flow of air at 2 L.min^−1^.

The measurements of Zrs were made during quiet sleep in the supine posture, with the head supported in a neutral position. A minimum of five technically acceptable 30-s data epochs was collected. Recordings (or short segments of them) that contained breath holds, cries, irregular breathing or leaks around the face mask were excluded. Zrs spectra were defined as reproducible if at least three of the spectra had Rrs values within 10% of each other. The individual Zrs spectra were evaluated by fitting a resistance (R)—compliance (C)—inertance (I) model to the measured data and the results averaged (Supplementary Figure S2). Resonance frequency (*f*res) was calculated as *f*res=1/(2π√CI).

### Collection of antenatal and early life data

Information regarding antenatal, birth and early life exposures, and events were collected by questionnaires at scheduled study visits.^16^ Maternal smoking was confirmed by a quantitative analysis of maternal urine cotinine, and all mothers underwent HIV testing at an antenatal visit and at birth; details of these tests are described in the online supporting information.

### Statistical analysis

Statistical analysis was performed using STATA 13 (STATA Corporation, College Station, Texas, USA). Data are presented as mean, standard deviation (SD), median and 25–75% and 95% confidence intervals (CI). The intra-subject coefficient of variation (CoV) was calculated as CoV = 100SD/mean for each infant's measurement. The relationships among anthropometric variables, prenatal/perinatal data and respiratory mechanics were examined using the Wilcoxon rank sum test for independent samples and Spearman's correlation analysis. A multivariate analysis examining the determinants of the Zrs parameters was undertaken, as detailed in the online supporting information. Differences at a *P*-value of <0.05 were considered statistically significant.

## Results

Of 219 infants tested, 42 infants who were born preterm or had previous pneumonia were excluded, leaving 177 healthy infants; 164 (93%) of whom had acceptable daa collected (see Supplementary Figure S1). The demographic data of the 177 infants are shown in Table 1.

**Table 1:** Demographics of infants (*n* = 177)

	African ethnicity *n* = 79 Median (25–75%)	Mixed African/other ethnicity *n* = 98 Median (25–75%)	Total *n* = 177 Median (25–75%)
Age (weeks)	7.7 (7.0; 8.1)	7.4 (6.7; 8.0)	7.6 (6.9; 8.1)
Weight (kg)	4.9 (4.6; 5.6)	4.7 (4.3; 5.3)	4.8 (4.4; 5.4)*
Weight for age z score	−0.2 (−0.7; 0.6)	−0.5 (−1.2; 0.3)	−0.36 (−0.98; 0.43)*
Length (cm)	56 (53; 57.8)	55 (53; 57)	55 (53; 57)
Length for age z score	−0.7 (−1.8; 0.2)	−0.7 (−1.8; 0.1)	−0.7 (−1.8; 0.1)
Gestational age (week)	39 (38; 40)	39 (38; 40)	39 (38; 40)
Birth weight (kg)	3.1 (2.9; 3.5)	3.0 (2.8; 3.5)	3.1 (2.8; 3.5)
Birth weight z score	−0.5 (−1.3; 0.04)	−0.8 (−1.5; −0.1)	−0.7 (−1.4; −0.04)
Birth length (cm)	50 (48; 53)	50.5 (48; 53)	50 (48; 53)
Birth length z score	0.0 (−0.8; 1.0)	0.1 (−1.1; 0.9)	−0.0 (−0.9; 0.9)

	***n* (%)**	***n* (%)**	***n* (%)**
Male	38 (48)	51 (52)	89 (50)
Maternal HIV (positive)	26 (33)	7 (7)	33 (19)*
Maternal smoking			
Active smoker	16 (20)	59 (62)	75(43)*
Passive smoke exposure	39 (50)	28 (30)	67 (39)*

(Associate Editor: Chi Chiu Leung).

* Statistically significant difference, *P* ≤ 0.05.

The values of impedance parameters R, C, I and *f*res are summarized in Table 2. The intra-subject variability, represented by the median (25–75%) of the intra-individual CoV, is also included. The single-frequency data at 8, 12 and 16 Hz are represented in Supplementary Table S1. The impedance parameters R and C, respectively, correlated well with the Rrs at 12 Hz and the effective compliance calculated from the Xrs values at 8 Hz (see Supplementary Figure S3). However, it should be noted that the intra-individual variability of R and C was considerably lower than that of the corresponding single-frequency Rrs and Xrs values, indicating the more robust nature of the model estimates. The Zrs parameters showed strong interdependences (see Supplementary Figure S4), with a negative correlation between C and R (r = −0.52; *P* < 0.001) and positive correlation between I and R (r = 0.57; *P* < 0.001).

**Table 2:** Respiratory impedance parameters (*n* = 164)

	Mean (SD)	Median (25–75%)	CoV (median (25–75%))
Resistance (cmH_2_O.s.L^−1^)	52.8 (19.1)	50.2 (39.5; 60.6)	5.9 (3.6; 9.5)
Compliance (mL.cmH_2_O^−1^)	0.85 (0.42)	0.78 (0.61; 0.99)	13.9 (6.9; 22.1)
Inertance (cmH_2_O.s^2^.L^−1^)	0.073 (0.044)	0.062 (0.050; 0.086)	14.0 (7.7; 24.0)
Resonance frequency (Hz)	23.7 (6.4)	22.1 (19.5; 26.5)	7.5 (4.1; 11.9)

CoV, intra-subject coefficient of variation.

**Figure 2:**
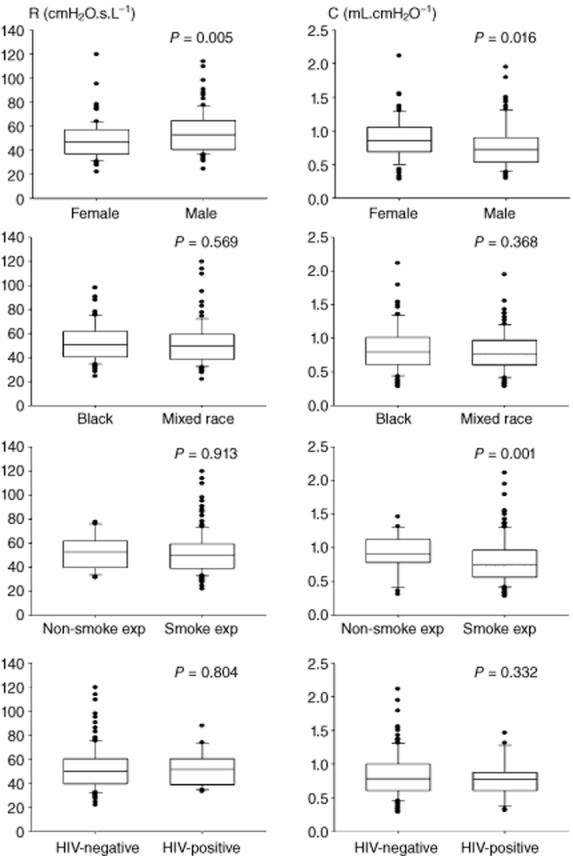
Resistance (R) and compliance (C) by sex, ethnicity, maternal smoke exposure and maternal HIV. Data are shown as median, 25–75% and 95% confidence intervals.

The association of demographic factors and early life exposure and respiratory mechanical parameters measured with FOT are represented in Figure 2. Male infants had 10% higher R (53.2 (40.9–65.4) vs 48.3 (37.2–58.8) cmH_2_O.s.L^−1^, *P* = 0.005) and 17% lower C compared with female infants (0.72 (0.53–0.9) vs 0.87 (0.69–1.08) mL.cmH_2_O^−1^, *P* = 0.004); I and *f*res were similar in male and female infants. Infants whose mothers smoked during pregnancy had a 21% lower C (0.71 (0.54–0.96) vs 0.92 (0.80–1.20) mL.cmH_2_O^−1^, *P* = 0.005) and 19% higher *f*res (24.6 (20.2–28.6) vs 20.6 (18.3–23.2) Hz, *P* = 0.007) compared with infants whose mothers did not smoke; with no difference between groups for R and I. Ethnicity and maternal HIV infection did not have a significant effect on any impedance measures.

Results of the multivariate analysis of respiratory impedance determinants are displayed in Supplementary Table S2. Adjusted for body size, ethnicity, sex and maternal HIV status, infants whose mothers smoked during pregnancy had 0.35 mL.cmH_2_O^−1^ lower C (95% CI: −0.55 to −0.15, *P* = 0.001) and 5 Hz higher *f*res (95%CI 1.9 to 8.1, *P* = 0.002) compared with infants whose mothers did not smoke. Infants whose mothers were exposed to passive cigarette smoke during pregnancy also had reduced C, 0.23 mL.cmH_2_O^−1^ lower (95%CI −0.41 to −0.04, *P* = 0.016) and 2.9 Hz higher *f*res (95% CI: 0.09–5.7, *P* = 0.04) compared with infants whose mothers were not exposed to passive smoke, although this effect was not as strong as the exposure to active maternal smoking. None of the exposures affected R or I measured with the FOT in the first few weeks of life.

## Discussion

This is the first study to report the use of FOT in unsedated healthy term infants 1–3 months of age. The success rate of 93% is higher than has been described in other infant cohorts reporting lung mechanics measurements in unsedated infants.^17^–^20^ The intra-test variability, ranging from 4% to 10% for resistance and from 7% to 22% for compliance, is similar to those reported in newborn infants^21^ and older children^22^ tested with the FOT. This suggests that FOT may be an appropriate lung function test for longitudinal studies through early childhood to adolescence. Further, this is the first investigation to report successful use of this technique in infants in a low- to middle-income setting, which carry a large burden of respiratory disease globally.^23^

Direct comparisons between resistance and compliance measured with FOT and that measured by other techniques in healthy unsedated infants, such as the single occlusion technique (SOT),^24^–^26^ cannot be made. In particular, the compliance obtained from the FOT measurements at frequencies much higher than the spontaneous breathing rate is approximately five times lower than the quasi-static compliance measured by the SOT.^9^,^26^ Since the respiratory system resistance is less frequency-dependent, the values of R determined in the present study with the FOT are similar to those obtained with the SOT. The R measured with FOT was slightly higher than that measured in unsedated European infants using the interrupter technique,^17^ which may be partially explained by the differences in measurement technique used but may also reflect the different populations with differing risk of exposure in these studies. The FOT has the advantage of measuring Zrs during uninterrupted normal breathing pattern without the need of respiratory pauses, and hence possibly a more relevant measure of respiratory mechanics during tidal breathing.^27^

The strong inverse relationship observed between R and C (Supplementary Figure S3) reflects the size effects on the resistive and compliant mechanical properties, while the positive correlation between R and I indicates the large contribution of the upper airways to the respiratory impedance. Male infants in the present study had a higher R and lower C as a group, compared with the female infants. This is consistent with previous studies that have shown male infants to have reduced early life lung function as compared with female infants.^26^,^28^–^32^ Hanrahan *et al*. investigated healthy infants at 2–6 weeks with SOT and reported that male infants had a significantly higher R (0.083 vs 0.079 cmH_2_O.s.mL^−1^; *P* = 0.003) but a non-significantly lower C (5.4 vs 5.54 mL.cmH_2_O^−1^; *P* = 0.37) compared with female infants.^26^ Stocks *et al*. tested preterm infants at 5-39 days with the SOT and multiple occlusion technique; female infants had a lower (although statistically not significantly different) resistance than males and significantly higher time to peak expiratory flow over total expiratory time.^28^ These sex differences in lung function early in life suggest that infant boys may have a less mature respiratory system at birth compared with girls. However, it has also been suggested in previous reports that postnatal growth and maturation may be faster in boys,^26^,^28^ which underlines the need for further investigations on the longitudinal changes of respiratory mechanics.

The relationship between maternal smoking and low infant lung function in early life is well established.^33^ Forced expiratory flow was shown to be lower in infants whose mothers smoked during pregnancy;^32^,^34^,^35^ additionally, respiratory compliance measured with SOT was reduced in infants at birth who had been exposed to *in utero* tobacco smoke compared with infants without exposure (3.6 vs 4.8 mL.cmH_2_O^−1^; *P* < 0.001, 95% CI: 0.89–1.55).^36^ The present cohort had a very high prevalence of maternal smoke exposure; 43% of infants studied had mothers who smoked during pregnancy, and a further 39% of babies had mothers who were exposed to environmental tobacco smoke. This is consistent with the low socioeconomic status of the cohort, where most infants live in overcrowded conditions. Infants of mothers who smoked during pregnancy in the present study had a significantly lower C compared with infants whose mothers did not smoke. The lack of effect on R in smoke-exposed infants is likely due to the fact that the upper airways contribute a significant proportion to total resistance and may mask a mild smoking-related change in lower airway resistance. Therefore, it remains unclear whether the resistive and elastic properties of the lung periphery were similarly affected by the smoke exposure or were dissociated in their responses.

Thirty-three (19%) infants had HIV-infected mothers; all infants completed the prevention of mother-to-child transmission (PMTCT) programme and no infants were found to be HIV-infected. HIV-exposed but uninfected infants have a higher incidence of wheezing illness and respiratory infections in early life,^37^ and have an increased risk of pneumonia with treatment failure, compared with unexposed uninfected infants.^38^ Whether this is due to increased risk factor exposure, impaired immunity or effect of HIV exposure on early lung growth is not known. In this cohort, maternal HIV exposure had no effect on respiratory impedance at 6 weeks of age, suggesting that low lung function at this early stage may not be the reason for increased pneumonia risk in early childhood; however, the data need to be interpreted with caution due to the small number of infants included and the exclusion of those with previous pneumonia. In addition, this cohort represents a group of HIV-infected woman in relatively good health, since they all received antiretroviral therapy as part of the PMTCT programme.

This study has shown that FOT is a useful test in the measurement of lung mechanics in unsedated healthy infants. FOT already has clinical application in preschool-aged and older children.^3^ Being able to measure respiratory impedance from early life through childhood to adolescence would allow tracking of lung growth and disease and monitoring of response to interventions. However, before FOT can be used routinely as an infant lung function test, normal variability, repeatability and data on bronchodilator response in acute and chronic respiratory diseases need to be established.

In conclusion, the present adaptation of the FOT is sensitive enough to detect the influences of sex and maternal tobacco exposure on respiratory mechanics in healthy unsedated infants. Further longitudinal studies of the impact of early life environmental exposures on lung development and growth will be important in defining risk factors for respiratory disease.

## Abbreviations

CI: confidence interval
COV: coefficient of variation
FOT: forced oscillation technique
PMTCT: prevention of mother-to-child transmission
Rrs: respiratory resistance
SD: standard deviation
SOT: single occlusion technique
Xrs: reactance

## Supporting Information

Additional Supplementary Information can be accessed via the html version of this article at the publisher's web-site:

**Supplementary Appendix S1** Additional information on study population, data collection and analysis. **Supplementary Table S1** Respiratory system resistance (Rrs) and reactance (Xrs) at 8, 12 and 16 Hz. **Supplementary Table S2** Multivariate analysis of lung function, anthropometry, gender, ethnicity, maternal HIV infection and maternal smoking. **Supplementary Figure S1** Chart of the study population and exclusions. **Supplementary Figure S2** Schematics of the model fitting to the Zrs spectra represented by the real part or resistance (Rrs, top) and imaginary part or reactance (Xrs, bottom). Left: fitting the Xrs data in the 8- to 48-Hz range; right: curtailed frequency range for Xrs data with resonance frequency <20 Hz. **Supplementary Figure S3** Respiratory system resistance (Rrs) at 12 Hz vs mean resistance (R) from model fitting (top); effective compliance calculated from the reactance at 8 Hz vs compliance (C) from model fitting (bottom). Lines of identity are shown. **Supplementary Figure S4** Relationships between the compliance (C, top) and inertance (I, bottom) and the resistance (R) of the total respiratory system. Symbols correspond to the mean values from each infant's measurement.
