# Supplementary material for: Respiratory impedance in healthy unsedated South African infants: Effects of maternal smoking
Source: Respirology. 2015 Jan 11;20(3):467–73. doi: 10.1111/resp.12463 (PMC4670479; doi:10.1111/resp.12463)
Supplement: Supplementary file 1 [file resp0020-0467-sd1.docx]

**Supplementary Information**

**Respiratory impedance in healthy unsedated South-African infants: effects of maternal smoking**

*Diane Gray, Dorottya Czövek, Emilee Smith^,^ Lauren Willemse, Ane Alberts, Zoltán Gingl, Graham L. Hall, Heather J. Zar, Peter D. Sly, Zoltán Hantos*

^1^Department of Paediatrics and Child Health, Red Cross War Memorial Children’s Hospital, University of Cape Town, Cape Town, South Africa

^2^Department of Medical Physics and Informatics, University of Szeged, Szeged, Hungary

^3^Queensland Children's Medical Research Institute, University of Queensland, Brisbane, Australia

^4^Center for Infectious Disease Epidemiology and Research, University of Cape Town, Cape Town, South Africa

^5^Department of Technical Informatics, University of Szeged, Szeged, Hungary

^6^Telethon Kids Institute, University of Western Australia, Perth, Australia

**Study population**

Healthy infants between 6 and 10 weeks of age enrolled in the Drakenstein Child Health study (DCHS),^1^ were eligible for the lung function measures. The DCHS study is a multidisciplinary birth cohort investigating the epidemiology and etiology of childhood respiratory illness and the determinants of child respiratory health in a peri-urban area in South Africa. The primary aim is to investigate aetiology, progression and risk factors for childhood pneumonia and the impact on child health. The DCHS is located in the Drakenstein area in the town of Paarl, a peri-urban area, 60 km outside Cape Town, South Africa with a population of approximately 200,000. The local economy is based around commercial agriculture and light industry. More than 90% of the population access health care in the public sector including antenatal and child health services. The public health system is comprised of 23 primary health care clinics and one centralized hospital, Paarl Hospital, where all births and all hospital based pediatric care, including admissions occur. Similar to many low and middle income countries, the area has a high burden of childhood diseases and pneumonia ^2^ and a high prevalence of risk factors associated with pneumonia or severe disease, such as tobacco smoke exposure, malnutrition or poverty. Pregnant women were recruited from two primary health care clinics: Mbekweni, of predominantly African ethnicity and Newman predominantly of mixed African/other ethnicity. This is a peri-urban, accessible, low socioeconomic community. Mother-infant dyads were enrolled at 20-28 weeks’ gestation and will be followed until children reach 5 years of age.

Lung function was tested at 6 weeks of age and is scheduled for 1 year of age and annually until 5 years. The first 219 infants presenting for FOT testing at the 6-week lung function visit were included in the current study. Of these, infants born premature (<37 weeks) and those who had previously had a lower respiratory tract infection were excluded from the current study. A further 13 infants were excluded for other reasons detailed in Fig. S1. The study was approved by the Faculty of Health Sciences, Human Research Ethics Committee, The University of Cape Town (401/2009) and by the Western Cape Provincial Health Research Committee. Mothers gave informed, written consent in their first language for their infants to participate.

**Collection of antenatal and early life data**

Maternal smoking was confirmed by a quantitative analysis of maternal urine cotinine (IMMULITE 2000 Nicotine Metabolite analyser, Siemens, Los Angeles, USA). Maternal urine specimens were collected antenatally and at birth. Smoking exposure based on maternal urine cotinine was defined as follows: active smoker, urine cotinine level of >500 ng.mL^-1^; passive smoker, 11- 500 ng.mL^-1^ and non-smoker <10 ng.mL^-1^.^3^ Following public sector protocol, all mothers would have had an HIV test at antenatal booking visit and repeated at delivery. Testing is done by rapid HIV immunoassay (Determine® HIV-1/2, Abbott, Illinois, USA) with a confirmatory ELISA test if the rapid test is positive (Enzygnost® Anti-HIV 1/2 Plus, Siemens Healthcare Diagnostic Products, Marberg, Germany). All HIV-exposed children had an HIV PCR test (Cobas Ampliprep system, Roche Molecular Systems, New Jersey, USA) done by 6 weeks of age.

**Model analysis of impedance**

Respiratory mechanical parameters. From the measurements of total respiratory impedance (Zrs) the parameters of respiratory mechanics were evaluated by fitting a resistance (R) – compliance (C) - inertance (I) model to the measured data (Figure S2). Resonance frequency (*f*res), the zero crossing of the imaginary part of Zrs (Xrs), was calculated as *f*res=1/(2π√CI). R was obtained as the mean value of R between 12 and 32 Hz; the R values at 8 Hz were omitted from the estimation of R because of systematically higher values reflecting the contribution of tissue resistance in these young infants.^4^ The R values at >32 Hz were also excluded because of occasional elevations in R as a consequence of observed distortion of the parabolic airflow profile.^5^ In order to obtain a balanced contribution from the elastic and inertive properties of Xrs to the fitting, C and I were estimated from the Xrs data between 8 and 32 Hz whenever *f*res was ≤20 Hz and between 8 and 48 Hz otherwise.

**Comparison of Zrs values and model parameters**

Table S1 summarizes the mean values of Rrs and Xrs data at 8, 12 and 16 Hz. The intra-individual coefficients of variation (CoV) were higher for all single-frequency estimates for Rrs (the median values were 8.8, 7.4 and 7.5% at 8, 12 and 16 Hz, respectively) than for averaged R (5.9%). The effective compliance (Crs,eff) calculated as Crs,eff=-1/(2π.f.Xrs) at 8 Hz was also more variable than C (median CoV: 21.4 vs.13.3%). The relationships between Rrs at 12 Hz and R (Figure S3, top), was very strong (r=0.89), with systematically higher values for Rrs, reflecting the initial negative frequency dependence of Rrs (see Table S1). The overestimation of C by the Crs,eff at 8 Hz increases progressively with C (Figure S3, bottom), since the positive contribution of the inertial reactance to Xrs is more appreciable when the lung compliance is higher.

**Multivariate analysis of determinants of impedance parameters**

The association between the Zrs parameters and anthropometric indices, ethnicity, gender; exposure to active and passive maternal smoking and maternal HIV infection were tested in a multivariate linear regression model. Factors showing significant association at a level of p=0.1 on initial univariate analysis (weight, gender and smoke exposure) and possible confounders (height, ethnicity, maternal HIV infection) were included in a multivariate linear regression model. The data are presented as coefficients, 95% CI and corresponding p-values in Table S2. Interactions were examined through stratification by gender. No evidence of interaction effects between covariates was found. However the study was underpowered for a comprehensive stratification analysis as subgroups were very small in sample size, but this was not the primary aim of the analysis.

**References**

1 Zar HJ, Barnett W, Myer L, Stein DJ, Nicol MP. Investigating the early-life determinants of illness in Africa: the Drakenstein Child Health Study. Thorax. 2014.

2 Ehrlich RI, Du Toit D, Jordaan E, Zwarenstein M, Potter P, Volmink JA, Weinberg E. Risk factors for childhood asthma and wheezing. Importance of maternal and household smoking. Am J Respir Crit Care Med. 1996; **154**: 681-8.

3 Diagnostics SMS. 2006. IMMULITE 2000, Nictine Metabolite. <http://www.medical.siemens.com/siemens/en_GLOBAL/gg_diag_FBAs/files/package_inserts/immulite_2000/Other_Analytes>. Accessed: February 2014.

4 Bates JH, Irvin CG, Farre R, Hantos Z. Oscillation mechanics of the respiratory system. Comprehensive Physiology. 2011; **1**: 1233-72.

5 Finucane KE, Dawson SV, Phelan PD, Mead J. Resistance of intrathoracic airways of healthy subjects during periodic flow. J Appl Physiol. 1975; **38**: 517-30.

6 *WHO Child Growth Standards*. Geneva: World Health Organisation, 2006.

**Table S1: Respiratory system resistance (Rrs) and reactance (Xrs) at 8, 12 and 16 Hz.**

| Frequency (Hz) | 8 | 12 | 16 |
| --- | --- | --- | --- |
| Rrs (cmH_2_O.s.L^-1^) Mean (SD) | 62.2 (22.1) | 58.9 (21.7) | 54.6 (20.9) |
| Xrs (cmH_2_O.s.L^-1^) Mean (SD) | -22.4 (12.2) | -15.5 (8.7) | -9.1 (7.2) |

**Table S2: Multivariate analysis of lung function, anthropometry, gender, ethnicity, maternal HIV infection and maternal smoking.**

| **RESISTANCE (cmH_2_O.s.L^-1^)** | | | | | | |
| --- | --- | --- | --- | --- | --- | --- |
|  | **Coefficient** | | **95% CI^1^** | | **p-value** | |
| Weight-for-age z score^2^ | 4.23 | 0.48 | | 7.99 | | 0.027 |
| Length-for-age z score | -0.99 | -3.76 | | 1.77 | | 0.479 |
| Male | 8.62 | 2.60 | | 14.64 | | 0.005 |
| Ethnicity - African | -2.05 | -9.14 | | 5.04 | | 0.569 |
| HIV | 1.06 | -7.37 | | 9.49 | | 0.804 |
| Smoking - Passive | -0.47 | -9.02 | | 8.07 | | 0.913 |
| Smoking - Active | 0.35 | -8.96 | | 9.67 | | 0.941 |
| **COMPLIANCE (mL-cmH_2_O^-1^)** | | | | | | |
|  | **Coefficient** | | **95% CI** | | **p-value** | |
| Weight-for-age z score | -0.018 | -0.099 | | 0.063 | | 0.667 |
| Length-for-age z score | 0.002 | -0.058 | | 0.061 | | 0.959 |
| Male | -0.161 | -0.291 | | -0.030 | | 0.016 |
| Ethnicity - African | -0.070 | -0.223 | | 0.083 | | 0.368 |
| HIV | -0.090 | -0.272 | | 0.093 | | 0.332 |
| Smoking – Passive | -0.227 | -0.412 | | -0.042 | | 0.016 |
| Smoking - Active | -0.352 | -0.553 | | -0.150 | | 0.001 |
| **INERTANCE (cmH_2_O.s^2^.L^-1^)** | | | | | | |
|  | **Coefficient** | | **95% CI** | | **p-value** | |
| Weight-for-age z score | 0.007 | -0.002 | | 0.015 | | 0.144 |
| Length-for-age z score | 0.000 | -0.006 | | 0.007 | | 0.971 |
| Male | 0.006 | -0.008 | | 0.021 | | 0.367 |
| Ethnicity - African | -0.010 | -0.027 | | 0.006 | | 0.219 |
| HIV | 0.002 | -0.017 | | 0.022 | | 0.819 |
| Smoking - Passive | -0.010 | -0.030 | | 0.010 | | 0.306 |
| Smoking - Active | -0.010 | -0.032 | | 0.012 | | 0.375 |
| **RESONANCE FREQUENCY (Hz)** | | | | | | |
|  | **Coefficient** | | **95% CI** | | **p-value** | |
| Weight-for-age z score | -0.831 | -2.068 | | 0.406 | | 0.186 |
| Length-for-age z score | -0.145 | -1.056 | | 0.766 | | 0.753 |
| Male | 1.171 | -0.811 | | 3.153 | | 0.245 |
| Ethnicity - African | 1.414 | -0.922 | | 3.750 | | 0.234 |
| HIV | 0.042 | -2.734 | | 2.818 | | 0.976 |
| Smoking - Passive | 2.901 | 0.086 | | 5.716 | | 0.044 |
| Smoking - Active | 5.016 | 1.948 | | 8.084 | | 0.002 |

^1^ 95% confidence intervals; ^2^ weight and height z-scores based on the WHO Child Health Standards ^6^

**Figure S1**.


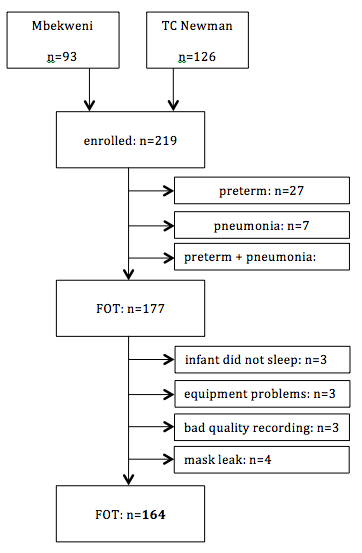


Chart of the study population and exclusions.

**Figure S2**


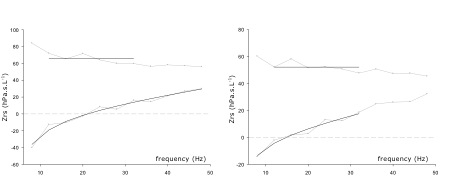


Schematics of the model fitting to the Zrs spectra represented by the real part or resistance (Rrs, top) and imaginary part or reactance (Xrs, bottom). Left*:* fitting the Xrs data in the 8-48-Hz range; right: curtailed frequency range for Xrs data with resonance frequency < 20 Hz.

**Figure S3**


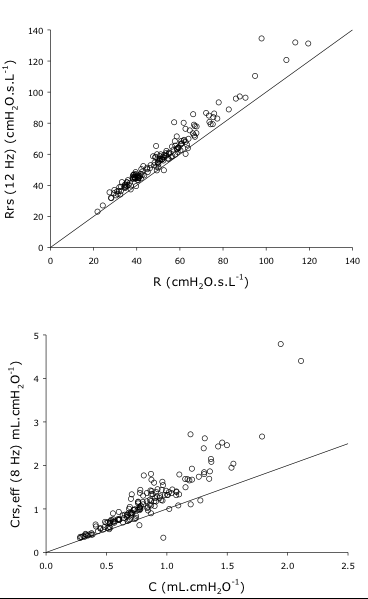


Respiratory system resistance (Rrs) at 12 Hz vs. mean resistance (R) from model fitting (top); effective compliance calculated from the reactance at 8 Hz vs. compliance (C) from model fitting (bottom). Lines of identity are shown.

**Figure S4**


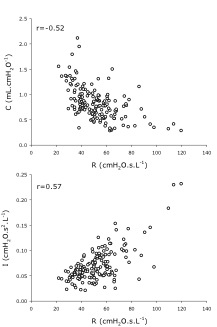


Relationships between the compliance (C, top) and inertance (I, bottom) and the resistance (R) of the total respiratory system. Symbols correspond to the mean values from each infant’s measurement.
